# Phrenology

**Published:** 1847-03

**Authors:** 


*Phrenology.*—It is well known that, among other arguments by which Gall upheld his view of the function of the cerebellum, he maintained that the size of this organ is less in castrated animals than in those possessing their sexual organs entire. He does not, however, appear to have made any very exact observations in support of this position, which he principally rested on a comparison of crania. Subsequent observers have not confirmed the statement. Even M. Vimont admits that the result of his experiments was doubtful. Desirous to put the question to the test, M. Leuret caused his former collaborateur, M. Lassaigne, (of whose qualifications as a scientific observer Mr. Noble expresses his ignorance,) to weigh the cerebra and cerebella of a sufficient number of stallions, mares, and geldings, and compare the results. A table of these weights, with that of the medulla oblongata, is given by M. Leuret, for every one of ten stallions, twelve mares, and twenty-one geldings; and the result is that the cerebellum of the gelding is, on the average, *both absotutely and relatively larger* than that either of the stallion or the mare. The manner in which these results are treated by Mr. Noble is, we are constrained to say, rather too characteristic of the general mode in which phrenologists treat evidence that does not suit their purpose. In the first place, not having seen the entire tables, but only the summary of them given in Dr. Carpenter's "Human Physiology," he makes objections which a reference to the tables would prove to be unfounded. His especial ground of refusal to admit the validity of these results, is the smallness of the number of observations, and the consequent uncertainty of the averages; but if he will refer to the tables themselves, he will see that even the *extremes* are as remarkable for the evidence they afford as are the *means.* Thus, the weight of the cerebellum of the stallion is to that of the cerebrum in the stallion, as from 1 : 7·46 in the *lowest*, to 1 : 6·25 in the *highest*, the *average* being 1 : 7·07. In the mare, the proportional weight of the cerebellum is greater, not from the larger absolute size of that organ, (as Mr. Noble would make out M. Leuret to assert, contrary to what he states is the invariable fact), but from the smaller size* of the cerebrum; it varies from 1 : 6·93 in the *lowest*, to 1 : 6·08 in the *highest*, the *average* being 1 : 6·59. In the gelding, the proportion varies from 1 : 6·44 in the *lowest*, (excluding one case in which the cerebrum was so enormously developed as to reduce the proportional weight of the cerebellum to 1 : 7·44, although the latter organ surpassed, in *absolute* weight, any other which is here recorded,) to 1 : 5·16 in the *highest*, the *average* being 1 : 5·97. We cannot but think that any unprejudiced person, on comparing these results, must admit that they are sufficient, at any rate, to countervail the vague statements of Gall, even though they may not be sufficiently numerous to establish an exact average of the relation. Phrenologists have too often shown a great horror of *precise* comparisons; but we submit that here is a case in which there can be no serious mistake, the cerebellum and cerebrum being so completely isolated by Nature that no accident or partiality on the part of the experimenter can affect the results in any considerable degree. We are suprised that a man of Mr. Noble's general candor and benevolence of disposition should have descended to the unworthy but easy method of invalidating the results of experiments which do not accord with his hypothesis, by imputing intentional deceit to M. Leuret, as being a known opponent of phrenology. It will be quite time, we submit, to do this, when Mr. Noble, or any other phrenologist, shall have brought before the world a more extensive series of observations made on the same plan, by which M. Leuret's results shall be overthrown. And even then it will be, of course, for the world to judge, from their relative estimate of the two parties, whether the system-makers or the objectors are most likely to be in error. The results of M. Leuret's inquiries have now been before the public for some years; but as we have not heard that they have been thus invalidated, we cannot but admit them as affording important negative evidence with regard to the phrenological view of the functions of the cerebellum.

We have left ourselves no space to touch upon the bearings of pathological observation, with regard to the two views of the functions of the cerebellum. This, however, we the less regret, since the very small number of well-observed cases at present recorded does not afford any decided evidence either

* We have discovered an error of calculation in M. Leuret's table, in regard to the weight of the cerebrum in Mare No. 7. The number (deduced from the weight of the entire encephalon) ought to be 436, instead of 336, and the proportion 6·60, instead of 5·09.

way; it being quite possible, however, to put this evidence in a form that is very favorable to either side, by neglecting all that bears the other way. This Mr. Noble has done in favor of the phrenological view; recording a small number of coincidences between injuries and diseases of the cerebellum, and disorder of the sexual organs, (among others, quoting a case in which the injury was a *flesh wound* in the neck, and in which there was no proof whatever of the cerebellum being affected,) but passing over with very imperfect notice the cases in which there was no such coincidence, or in which the phenomena tended the other way. He gives in detail, however, two cases recorded by Dr. Cowan, which seem to have had some influence with that gentleman—a professed phrenologist, be it observed, but at the same time a man of too philosophical a mind to be a blind partisan of the system,—in leading him to view with more favor the doctrines of Flourens. This forfeiture of allegiance to Gall, on the part of one of his followers, very amusingly excites Mr. Noble's surprise; his conviction of the truth of the system being such, that he cannot understand how any one who has once embraced it can relinquish one tittle of its dogmata. And he devotes some space to the analysis of the details of Dr. Cowan's cases, with the view of bringing them into harmony with Gall's doctrine; in this, however, he seems to us to display more ingenuity than philosophy.

On the whole, then, we are disposed to infer, from the remarkable concurrence between the results of experiment and the data supplied by comparative anatomy, that one function of the cerebellum is to regulate and combine the muscular movements required to perform a large number of our voluntary actions. And if the evidence, furnished by observation of the human species only, should prove to be such as to establish a constant relation between the development of this organ in him and the strength of his sexual propensity, (a position we by no means dispute, our argument being only directed against the insufficiency of the evidence on which it rests at present,) we must regard this as a *superadded* function, not as the fundamental purpose of the organ.

We conclude as we commenced, by disclaiming any hostility whatever to the phrenological system in the abstract; and by freely admitting the *general* coincidence between the indications of human character which are afforded by cranioscopical examination and those derived from a direct acquaintance. But we consider that in building up their system, the followers of Gall have been too disregardful of evidence supplied from other sources than observation of man; and that they have been misled, as to the fundamental question of the connection of the cerebrum with the purely instinctive actions, by their inattention to comparative anatomy, which proves that the cerebrum *cannot* be the instrument of those actions; and have glossed over the important objection which the nondevelopment of the posterior lobes in the lower mammalia and in all the oviparous vertebrata interposes to the location of the animal propensities in them. So far, however. from availing ourselves of these errors, as conclusive arguments against the whole system, we have endeavored, by a new analysis of the propensities and emotions, to show that the facts supplied by comparative anatomy may be brought into conformity with the physiology of Gall; and that the phrenological system may be planted upon a much more secure and extended basis than it has yet possessed; a new and more exact series of observations, however, being required to build it up with anything like firmness and consistency. We cannot regard the question of the functions of the cerebellum as at all fundamental in its character; and can easily understand how a candid phrenologist like Dr. Cowan may, on this point, embrace the views held, we believe, by all the leading physiologists of the day.—*Brit. and For. Med. Review.*

